# Severe pan-uveitis in a patient treated with vemurafenib for metastatic melanoma

**DOI:** 10.1186/1471-2407-13-561

**Published:** 2013-12-01

**Authors:** Sanne EJ Wolf, Christina Meenken, Annette C Moll, John B Haanen, Michiel S van der Heijden

**Affiliations:** 1 Department of Ophthalmology, VU University Medical Center, Amsterdam, The Netherlands; 2 Department of Medical Oncology, Netherlands Cancer Institute, Plesmanlaan 121, 1066 CX Amsterdam, The Netherlands

**Keywords:** Melanoma, Uveitis, Vemurafenib, BRAF

## Abstract

**Background:**

Vemurafenib, an inhibitor of genetically activated BRAF, is now commonly prescribed for metastatic melanoma harboring a *BRAF* mutation. Reports on side effects have focused on cutaneous complications. We here present a case of a severe pan-uveitis associated with vemurafenib use.

**Case presentation:**

A 63-year old female was treated with the BRAF inhibitor vemurafenib for metastatic melanoma. After seven weeks of treatment, she developed near-complete visual loss in the course of a few days, as a result of severe uveitis. Vemurafenib had to be discontinued and systemic and topical corticosteroids were initiated. The visual symptoms improved slowly, however the cerebral metastases progressed and the patient died from her disease.

**Conclusion:**

Treatment with vemurafenib has become an important component of standard clinical care for patients with metastatic melanoma. In addition, it is one of the best examples of genotype-directed therapy. This case illustrates that vemurafenib-induced uveitis can develop fast and be slow to resolve. Awareness of this potentially severe side effect is of major importance to oncologists and aggressive treatment should be considered.

## Background

Until very recently, treatment options for metastatic melanoma were virtually non-existent. This situation has dramatically changed with the introduction of the BRAF inhibitors vemurafenib [1] and dabrafenib [2] and the anti-CTLA4 antibody ipilimumab [3,4]. Additionally, promising therapeutic strategies currently in phase 3 trials include combinatorial approaches of BRAF inhibitors with MEK inhibitors [5] and anti-PD1 and anti-PD-L1 antibodies [6]. Side effects of these new classes of therapeutics are very different from traditional chemotherapy, as has been particularly noted for ipilimumab [7]. Side effects of vemurafenib are generally of low to moderate severity and include arthralgia, rash, fatigue, photosensitivity and keratoacanthoma or squamous cell carcinoma of the skin [1]. We present a case of a patient on vemurafenib with near-complete visual loss caused by a pan-uveitis.

## Case presentation

A 63-year old female presented with weakness of her left leg. She had been treated in 2001 for a superficially spreading melanoma, Breslow depth 1.4 mm. Magnetic Resonance Imaging (MRI) of the brain revealed a metastasis in the right frontal lobe with signs of hemorrhage and several additional small cerebral metastases. Subsequent computed tomography (CT) scans showed metastases to the thoracic and lumbar spine. A biopsy of a metastasis at the sacro-iliac joint revealed melanoma cells; mutation analysis of the *BRAF* gene showed a V600E mutation in exon 15. Initial treatment consisted of whole-brain radiation (7×4 Gy), and radiation to the thoracic and lumbar spine. Since all of the known metastases had been treated with radiation, systemic treatment was not initiated yet.

**Figure 1:**
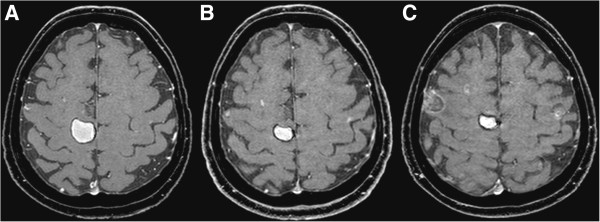
**Gadolinium-enhanced T1-weighted magnetic resonance images of cerebral metastases. A**. MRI of the brain before treatment with vemurafenib. **B**. MRI at presentation with visual loss. **C**. MRI four weeks after cessation of treatment.

A CT scan made two months later revealed new metastases in the right lung, peritoneum and left groin. The patient had recovered well from the cerebral hemorrhage and the treatment of her cerebral and spinal metastases. She was able to walk for a short distance and her only complaint was a moderate hearing loss. MR imaging of the brain revealed a slight decrease of the cerebral hemorrhage and no new metastases (Figure 1A). Vemurafenib, an oral inhibitor of the BRAF kinase, was initiated at 960 mg bi-daily. Treatment was initially tolerated well except for mild periorbital edema.

**Figure 2:**
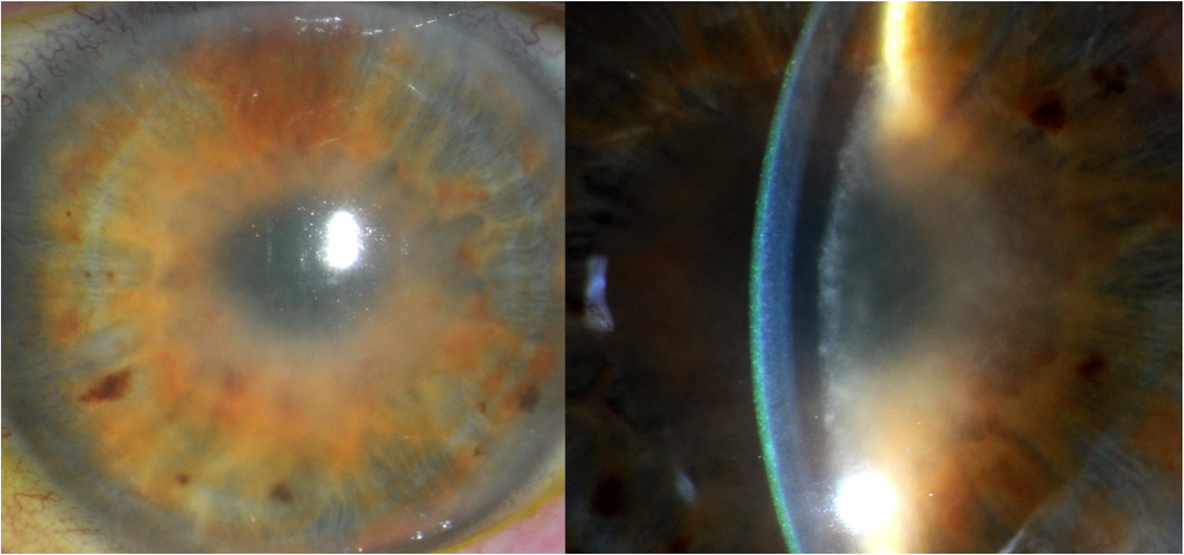
**Uveitis with cells in the shallow anterior chamber.** Slit lamp examination: shallow anterior chambers and a severe fibrinous and cellular reaction, covering the entire pupillary opening.

After seven weeks of treatment with vemurafenib, she presented to the hospital with severe visual loss, which had started several days earlier. She did not have a previous medical history of ocular problems. An MRI of the brain showed less hemorrhage of the right frontal metastasis and no increase in size of the other small cerebral lesions (Figure 1B). A CT scan showed regression of the peritoneal and pulmonary lesions and stabilization of the metastasis to the right groin. Ophthalmological examination revealed a visual acuity of only light perception in both eyes. Slit lamp examination showed shallow anterior chambers in both eyes, and a severe fibrinous and cellular reaction, covering the entire pupillary opening and causing a pupillary block and secondary elevation of the ocular pressure (Figure 2). Ultrasound imaging of the eyeball showed signs of scleritis. Vemurafinib was considered the culprit and therefore discontinued; treatment with topical and systemic coricosteroids (prednisone, 60 mg per day) was initiated. The patient’s scleritis decreased and her vision improved slowly to a visual acuity of 0.25 in the right and 0.8 in the left eye. At that time, fundoscopic examination was possible, and did not reveal signs of vasculitis nor chororetinitis in both eyes. A surgical peripheral iridectomy was performed in the right eye to reverse a pupillary block caused by posterior synechiae.

Four weeks after cessation of treatment, she presented with progressive aphasia. An MRI of the brain showed progression of cerebral metastases with new hemorrhages in several metastases (Figure 1C). At that moment, her vision had improved, but had still not fully recovered. Because of the severe impact of the visual loss on quality of life, and since the response of the cerebral metastases at 7 weeks of treatment with vemurafenib showed stabilization at best, a second attempt of treatment with BRAF inhibitors was not initiated. Second line treatment with ipilimumab, an anti-CTLA4 antibody, was considered. The occurrence of a severe pan-uveitis was judged to be a contraindication to therapy that acts by stimulating the immune system. Additionally, she was still being treated with systemic corticosteroids. Dacarbazine was considered, but viewed as a treatment with little chance of response in this setting. The patient and her family preferred to refrain from further systemic treatment of her cancer. She died at her home six weeks later.

## Conclusions

We here present a case of severe vemurafenib-induced uveitis, with near-complete visual loss developing in the course of only a few days. Mild cases of uveitis have been noted in the original phase III trial [1] (product insert; 2,1%) and were reported in a recent poster abstract from an Australian ocular clinic in 23/516 (4.5%) of patients treated with vemurafenib [8]. These cases usually resolved with topical corticosteroids, while continuing vemurafenib. This is the first report to our knowledge of a vemurafenib-induced pan-uveitis leading to near-complete visual loss.

Uveitis is the process of intraocular inflammation and may result from different causes: infections, systemic immune-mediated disease, and masquerade syndromes. Pan-uveitis is defined as simultaneous inflammation in the anterior chamber, vitreous humor, and retina or choroid. Slit lamp and fundoscopic examination are necessary to establish the presence of uveitis. Drug-induced uveitis is a rare clinical condition [9]. A wide range of medications can cause drug-induced uveitis, as for example rifabutin. A recent review by London et al. [10] summarized that the underlying mechanism of drug-induced uveitis is still mostly unclear and that both inflammatory and toxic reactions may play a role. An immunologic cross-reaction between vemurafenib and antigens in the uvea could play a role, however this remains speculative.

Drug-induced uveitis is usually reversible within weeks of discontinuation of the offending drug. Noninfectious causes of anterior uveitis are in general treated with topical glucocorticoids several times a day. Oral glucocorticoids are reserved for patients with bilateral disease or for patients who do not respond to topical medications. The role of tumor necrosis factor-alpha (TNF-α) inhibitors in the treatment of patients with uveitis is being investigated [11]; these inhibitors appear to be more effective than corticosteroids in some patients with noninfectious uveitis [12]. In the case of metastatic melanoma, resolution of symptoms is pivotal to initiate a new line of treatment. This would argue in favor of aggressive treatment early in a case of severe uveitis, with systemic corticosteroids and possibly anti-TNF-α blockade.

Reports on side effects of vemurafenib have so far focused on cutaneous findings. One of the most concerning side effects is the development of cutaneous malignancies, primarily well-differentiated squamous cell carcinomas (SCC) and keratoacanthomas (KA), which occur in up to 25% of vemurafenib users [13]. Now that vemurafenib has become a component of the routine clinical treatment of metastatic melanoma, awareness of rare but severe side effects of this drug is of major importance to clinicians. This case illustrates a potentially severe ocular side effect in patients treated with vemurafenib.

## Consent

Written informed consent was obtained from the family of the patient for publication of this case report and the accompanying images. A copy of the written consent is available for review by the editor of this journal.

## Competing interests

Dr. Haanen reports being a member of the Roche advisory board for melanoma. The other authors report no competing interest.

## Authors’ contributions

All authors were involved in the clinical treatment of the patients and contributed writing the manuscript. All authors read and approved the final manuscript.

## Pre-publication history

The pre-publication history for this paper can be accessed here:

http://www.biomedcentral.com/1471-2407/13/561/prepub
